# Young man with painful black dots on left foot

**DOI:** 10.11604/pamj.2018.31.187.16029

**Published:** 2018-11-16

**Authors:** Fred Bernardes Filho, Loan Towersey

**Affiliations:** 1 Dermatology Division, Department of Medical Clinics, Ribeirão Preto Medical School, University of São Paulo, Ribeirão Preto, Brazil; 2 AIDS Division, Carlos Tortelly Municipal Hospital, Ministry of Health, Niterói, Brazil

**Keywords:** Foot Injuries, Cactaceae, Sports Medicine

## Image in medicine

A 30-year-old previously healthy man was admitted to the emergency room due painful black dots on the left foot. On physical examination, multiple erythematous macules with central black dots on left heel (A) were observed. After topical anaesthesia (lidocaine 40mg/g cream), several spines were removed. The patient was seen in a dermatology consultation complaining of local pain 22 days later. Dermatological examination revealed local erythema, and dermoscopy showed several punctiform wounds, some centered by dark brown pinpoint dots or dashes (B). The diagnosis of injuries caused by spicules of cactus in the heel was made. The removal of the fragments from the patient's foot and the follow-up visits to dermatologist led to a successful treatment that allowed the patient to return to work promptly. He was walking on an ecologic trail in Arraial do Cabo, Rio de Janeiro, when the pain started (C). Encounters with cactus spines, needles, prickles and glochids (barbed hairs) are rarely reported. Common signs of a retained cactus spine include: sharp pain as pressure is applied to the site, and discoloration of the skin. Smaller spines of the cactus plant break off easily on contact, and the fragments frequently get embedded in the skin and soft tissues. The lesion is predominantly aseptic. The most frequently affected area by cactus spine-related injuries are extremity and non-fluctuant tender skin papules which is the most frequently presented. Dermoscopy can be useful in helping to promptly detect and remove remaining embedded spines as seen in this cactus injury. Clinicians should consider injuries by cactus's spines in the differential diagnosis of retained foreign material.

**Figure 1:**
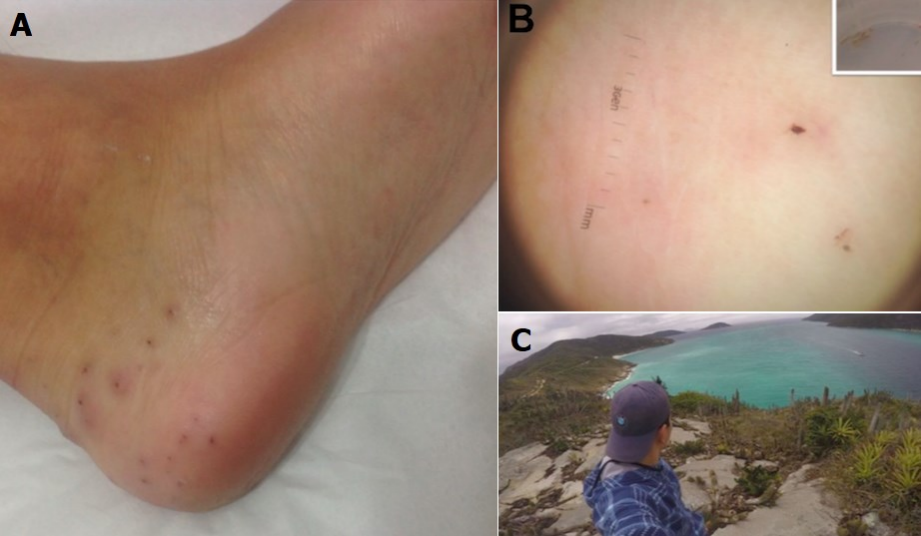
(A) multiple erythematous macules with central black dots on left heel; (B) dermoscopy showing punctiform wounds with dark brown spots surrounded by erythema (10x). Insert showing remaining cactus spines removed from the left heel; (C) distracted, walking on the rocks, at the end of an ecologic trail in Arraial do Cabo, Rio de Janeiro, Brazil, the patient stepped accidentally in cactus. Observe many cacti (Pilosocereus sp.) on the rocks, by the seaside, a semi-arid bioma (regional “caatinga”)

